# Ecophysiology, secondary pigments and ultrastructure of *Chlainomonas* sp. (Chlorophyta) from the European Alps compared with *Chlamydomonas nivalis* forming red snow

**DOI:** 10.1093/femsec/fiw030

**Published:** 2016-02-15

**Authors:** Daniel Remias, Martina Pichrtová, Marion Pangratz, Cornelius Lütz, Andreas Holzinger

**Affiliations:** 1 University of Applied Sciences Upper Austria, Wels, Austria; 2 Charles University in Prague, Faculty of Science, Department of Botany, Prague, Czech Republic; 3 University of Innsbruck, Institute of Pharmacy/Pharmacognosy, Austria; 4 University of Innsbruck, Institute of Botany, Austria

**Keywords:** astaxanthin, cryoflora, snow algae, spores, ultrastructure

## Abstract

Red snow is a well-known phenomenon caused by microalgae thriving in alpine and polar regions during the melting season. The ecology and biodiversity of these organisms, which are adapted to low temperatures, high irradiance and freeze–thaw events, are still poorly understood. We compared two different snow habitats containing two different green algal genera in the European Alps, namely algae blooming in seasonal rock-based snowfields (*Chlamydomonas nivalis*) and algae dominating waterlogged snow bedded over ice (*Chlainomonas* sp.). Despite the morphological similarity of the red spores found at the snow surface, we found differences in intracellular organization investigated by light and transmission electron microscopy and in secondary pigments investigated by chromatographic analysis in combination with mass spectrometry. Spores of *Chlainomonas* sp. show clear differences from *Chlamydomonas nivalis* in cell wall arrangement and plastid organization. Active photosynthesis at ambient temperatures indicates a high physiological activity, despite no cell division being present. Lipid bodies containing the carotenoid astaxanthin, which produces the red color, dominate cells of both species, but are modified differently. While in *Chlainomonas* sp. astaxanthin is mainly esterified with two fatty acids and is more apolar, in *Chamydomonas nivalis*, in contrast, less apolar monoesters prevail.

## Abstract (precis)

Red snow composed of *Chlainomonas* sp. was investigated with regard to its ecophysiology, secondary pigments and ultastructure, and several differences in astaxanthin composition and chloroplast arrangement were found when compared with *Chlamydomonas nivalis*.

## INTRODUCTION

Long-lasting, slowly melting summer snow fields in alpine and polar regions are the predominant habitats for extremophilic microalgae causing red snow or other colorations (Hoham and Duval 2001). Photoautotrophs adapted to these harsh conditions mostly belong to the Chlamydomonadales (Chlorophyta; Komárek and Nedbalová 2008). Snow algae are of general interest in terms of understanding fundamental cellular strategies to cope with low temperatures, extreme irradiation and desiccation stress due to freezing events. In addition, highly abundant compounds reducing or preventing abiotic stress might be of commercial interest (Varshney *et al.*^2015^). Commonly, populations causing red snow are connected with *Chlamydomonas* cf. *nivalis*, which is recognized as a collective taxon (Kol 1968). The typical cell morphology consists of spherical red spores that have a single-layered cell wall never detached from the protoplast, occasionally encapsulated by cryoconite particles (Remias, Lütz-Meindl and Lütz 2005). This taxon has been reported from almost all mountainous and polar regions worldwide, and recently even from snow at an African glacier in Tanzania (Uetake *et al.*^2014^), the last continent so far without reports of *Chlamydomonas* (*Cd.*) cf. *nivalis*—the first report of snow algae from Africa (Atlas Mountains), by Duval, Duval and Hoham (1999), did not mention this species. Other algae causing red snow belong to the genus *Chlainomonas*. These cells are usually larger than the first species and have an ellipsoidal shape; cell walls were earlier described as being removed from the protoplast or multilayered; swarmers have four flagella; and the cytoplasm is almost entirely occupied by red pigments (Ettl 1983). Two species of this genus that live in snow have been described, *Chlainomonas* (*Cn.*) *rubra* (Hoham 1974a) and *Cn. kolii* (Hoham 1974b). They have been found in the European Alps (Kol 1970), North America (Hoham 1974a,b), New Zealand (Novis 2002a), Spitsbergen (Kvíderová 2012) and Bulgaria (Cepák and Lukavský 2013). Using phylogenetic trees based on *rbc*L gene sequences, both Novis *et al.* (2008) and Muramoto *et al.* (2010) found *Chlainomonas* embedded in a clade of *Chloromonas* species that also live in snow habitats, despite the spores of *Chlainomonas* having morphological similarities to a different, *Chlamydomonas*-related clade of snow algae.

The abundant secondary pigment of all snow algae that have been investigated is the keto-carotenoid astaxanthin (and esterified derivatives), which is responsible for the red to orange appearance of these green algae, in particular of the lipid bodies in the cytoplasm (Remias, Lütz-Meindl and Lütz 2005; 2013; Řezanka *et al.*^2013^). The physiological role of astaxanthin is multifunctional: it protects the chloroplast against excessive irradiation and thus prevents photoinhibition or UV damage, it may serve as a powerful antioxidant, and it may represent a ‘metabolic sink’ of the non-dividing spores (Li *et al.*^2008^; Lemoine and Schoefs 2010). As astaxanthin is a nitrogen-free compound, its synthesis is not limited by low nutrients in the habitat, which is the case for snow remote from sea shores or any animal colonies (Hoham and Duval 2001). Further essential snow algal compounds are, for example, polyols like glycerol or ice-nucleating proteins, which lower the intra- or extracellular freezing point or prevent formation of damaging ice crystals within or close to the cells (Leya 2013; Raymond 2014). In contrast, the red pigments of *Chlainomonas* have never been studied before.

Global distribution and local abundances of *Chlainomonas* causing red snow seems to be restricted compared with the commonly reported *Cd.* cf. *nivalis*. We investigate here the hypothesis that these genera have different ecological demands. *Chlamydomonas* cf. *nivalis* is typically known from exposed seasonal snowfields above timberline, whereas *Chlainomonas* was found either in shaded areas below a canopy (Hoham 1974b) or in locations where snow becomes slushy during summer because meltwater drainage is limited (Remias 2012). This is common either in snow on glacier surfaces or in snow on the melting ice cover of high mountain lakes (Novis 2002b; this study). Another reason for a low number of reports could be misidentifications. For example, cell stages of the snow alga *Chloromonas* (*Cr.*) *bolyaiana* (Kol 1970) and *Cd. sanguinea* (Ettl 1983) have similar morphologies. Additionally, old spores of *Chlainomonas* turn from ellipsoidal to spherical during maturation and thus become almost identical to those of *Cd.* cf. *nivalis* (Hoham 1974a). In addition, the red cytoplasmic pigmentation is very similar in appearance to that of *Cd.* cf. *nivalis*.

Consequently, an intention of this study was to demonstrate specific characteristics of *Chlainomonas* spores at the light microscopy (LM) level, especially in contrast to *Cd.* cf. *nivalis.* Moreover, transmission electron microscopy (TEM) investigations were performed to visualize differences. Finally, chromatographic methods (liquid chromatography using chemotaxonomic characteristics) were used to test for different secondary pigment patterns. The ecophysiology of *Chlainomonas* has been mainly uninvestigated so far, and probably the only work was by Novis (2002b) for *Cn. kolii*. Therefore, a further aim of this study was to achieve insight into cellular adaption strategies. Field samples were harvested *in situ* mainly from snow slush on the melting ice cover of a high alpine lake in the Austrian Alps, where blooms of this species occur annually. We performed structural and ultrastructural studies, photosynthesis measurements and analysis of the secondary red pigmentation. The results were partly compared with red snow caused by *Cd.* cf. *nivalis* originating from the same region. For the latter, several physiological studies were previously reported (Stibal *et al.*^2007^; Lukeš *et al.*^2014^). Additionally, a ‘chemotaxonomic’ approach was undertaken for the first time with snow algae, comparing the patterns of native astaxanthin derivatives. We hypothesized that different taxa causing red snow, despite having a rather similar morphology, can be distinguished either by their ecological demands or by the polarity and molecular size of their individual astaxanthin esters. Moreover, we investigated apparent cytological differences.

## MATERIALS AND METHODS

### Field work and sample preparation

*Chlainomonas* sp. was collected from slushy red snow at the ice cover of Gossenkölle Lake (Kühtai valley, Tyrol, Austria) on 19 June 2008 (sample HW02), 12 June 2009 (AS02), 3 June 2010 (GK04), 15 June 2010 (GK12) and 11 June 2015 (DR67). A further sample was harvested from snow slush at Hallstätter glacier close to Mount Dachstein (Upper Austria) on 17 July 2009 (DR53). The complete list of *Chlainomonas* sp. collections with elevation, geographic position and snow chemistry is given in Table 1. *Chlamydomonas* cf. *nivalis* causing red snow was also collected in the Austrian Alps; a complete list with collection details is given in Supplementary Table 1. Before harvesting cells with a stainless steel shovel, a surface snow layer of approximately 1 cm, containing also allochthonous debris was removed. Then, snow was gently melted at 4°C and further cleaned of impurities by filtering through stainless steel sieves with 800, 400, 200 and 100 μm mesh size. The electrical conductivity of the meltwater was measured with a Cond 340i, and the pH with an Inolab system (both WTW Instruments, Germany). For TEM, field samples were further cleaned of microdebris by density centrifugation, using a gradient with layers of 0, 50 and 100% Percoll (Sigma-Aldrich) at 400 *g* for 10 min. For analytical purposes, the meltwater containing the cells was vacuum filtered through 47 mm glass fiber disks (Whatman GF/C). Filters with attached cells were rapidly frozen in liquid nitrogen and subsequently freeze-dried in a lyophilisator for 48 h. The lyophilized samples were stored at –50°C until analysis.

**Table 1.** Samples of red snow from the Austrian Alps containing *Chlainomonas* sp. with collection date, name of location, geographic position and altitude. Furthermore, pH and electrical conductivity of snow meltwater, as well as average cell sizes are given, if available (when only spherical cells present: diameter is stated as ‘length’).

Sample	Date	Location	GPS position	Altitude (m)	pH	EC (μS cm^−1^)	Length (μm)	Width (μm)
HW02	19 Jun 2008	Gossenkölle lake, T	N47°13.753 E11°00.863	2411	5.9	2.1	28–36	
AS02	12 Jun 2009	Gossenkölle lake, T	N47°13.753 E11°00.863	2411	5.8	10.0	32.7 ± 5.9	
GK04	3 Jun 2010	Gossenkölle lake, T	N47°13.753 E11°00.863	2411	6.0	6.9	n/a	n/a
DR53	17 Aug 2009	Hallstätter Glacier, U	N47°28.640 E13°36.927	2640	5.9	7.1	26.5 ± 3.8	22.2 ± 4.8
GK12	15 Jun 2010	Gossenkölle lake, T	N47°13.753 E11°00.863	2411	5.4	2.6	27.4 ± 4.9	23.3 ± 2.8
DR67	11 Jun 2015	Gossenkölle lake, T	N47°13.762 E11°00.885	2411	5.3	7.4	36.3 ± 3.5	33.1 ± 2.1

Abbreviations: EC, electrical conductivity; n/a, not available; T, Tyrol; U, Upper Austria.

### Light and transmission electron microscopy

For LM studies of ripening spores, field samples were kept alive in the laboratory for several weeks after collection at 4°C and approximately 100 μmol PAR m^−2^ s^−1^ (16 h d^−1^). Cells were observed by a Zeiss (Carl Zeiss AG, Jena, Germany) Axiovert 200M light microscope equipped with a Zeiss MRc5 camera with a ×63, 1.4 NA objective. Specimen temperature was kept at 1°C during observation to prevent heat stress in a thermoregulated chamber (LM-TCC; Buchner, Lütz and Holzinger 2007). Chlorophyll autofluorescence images were captured with a Zeiss 09 filter set (ex: 450–490 nm, em: 515 nm long pass (LP). Cell numbers per unit meltwater volume were obtained with a 0.5 mL plankton-counting chamber according to Kolkwitz (Hydro-Bios, Kiel, Germany). The TEM fixation of the sample HW02 and embedding protocol was applied according to Holzinger, Roleda and Lütz (2009). Briefly, samples were fixed in 2.5% glutaraldehyde in 10 mM caccodylate buffer (pH 7.0) for 2 h at 4°C, postfixed in 1% OsO_4_ overnight at 4°C, gradually dehydrated and embedded in modified Spurr's resin. Ultrathin sections were prepared by a Leica Ultramicrotome and counterstained, and samples were examined with a Zeiss Libra 120 TEM at 80 kV.

### DNA isolation, polymerase chain reaction and phylogenetic analyses

The field sample of *Chlainomonas* sp. DR67 was centrifuged and 200 μL of the Instagene Matrix (Bio-Rad Laboratories, USA) was added to the pellet. Then, the cells were mechanically damaged by shaking with glass beads in a Mixer Mill MM 400 (Retsch, Germany). The mixture was kept at 56°C for 30 min while being stirred, vortexed shortly and subsequently kept at 99°C for 8 min without stirring. After being shortly vortexed again, the samples were centrifuged at 17 000 g for 2 min and the supernatant was used as a polymerase chain reaction (PCR) template. The primers 7F and 1391R (Verbruggen *et al.*^2009^) were used for the amplification of the *rbc*L gene. Each PCR reaction contained 13.15 mL sterile Milli-Q water, 2 mL MgCl_2_ (25 mM), 2 mL AmpliTaq Gold 360 Buffer (Applied Biosystems, Carlsbad, CA, USA), 0.4 mL dNTP mix (10 mM), 1 mL of the 7F primer, 0.25 mL of the 1391R primer (primer concentration 25 pmol mL^–1^), 0.2 mL AmpliTaq Gold 360 DNA Polymerase and 1 mL DNA (10 ng μL^−1^). The PCR consisted of an initial denaturation for 3 min at 94°C, followed by 35 amplification cycles, each with a 1 min denaturation step at 94°C, 1 min annealing at 40°C and 2 min extension at 72°C, and a final extension stage at 72°C. for 5 min. The purification of the PCR product and DNA sequencing were performed by Macrogen Inc. (Seoul, South Korea). The newly obtained sequence was submitted to the European Nucleotide Archive (ENA) under the accession number LN897303. An 899 nucleotide alignment was created using additional *rbc*L sequences of 17 related strains and two outgroup strains, published in the GenBank database. Two different phylogenetic analyses were performed: Bayesian inference (BI) and maximum likelihood (ML). The sequence evolution model was determined as GTR + gamma by MrModelTest 2.3 (Nylander 2004) using the Akaike Information Criterion. The BI phylogenetic tree was constructed using MrBayes 3.2.1 (Ronquist and Huelsenbeck 2003). Two parallel Markov chain Monte Carlo runs were carried out for 3 000 000 generations, each with one cold and three heated chains. Convergence of the two cold chains was checked by the average standard deviation of split frequencies: the value was 0.000971. Trees and parameters were sampled every 100 generations, and trees from the initial 1000 generations were discarded using the sumt burnin function. Bootstrap analysis was performed by maximum likelihood (ML) in Garli 2.0 (Zwickl 2006) and PAUP* Portable version 4.0b10 (Swofford 2002). ML analyses consisted of rapid heuristic searches (100 pseudo-replicates) using automatic termination (genthreshfortopoterm command set to 100 000).

### Photosynthesis

Light-dependent oxygen production and respiration was measured 1 day after collection (sample GK04) in a temperature-controlled 3 mL acryl-chamber thermostated to 1°C, connected with a Fibox 3 optode and an oxygen dipping probe (PreSens, Germany) according to Remias *et al.* (2013). The average turnover per unit time of four replicates (*n* = 4) was normalized to the amount of chlorophyll a per sample. The chlorophyll a content was calculated after cell extraction with a PE Lambda 20 spectrophotometer (Remias *et al.*^2013^).

### Pigment characterization by HPLC

Freeze-dried algae were ground with a grinding mill with Teflon jars and quartzballs (Mikro-Dismembrator S, Sartorius, Germany) and dissolved in 3 mL *N*,*N*-dimethylformamide (Sigma-Aldrich) for apolar pigment extraction. Extracts were stored at –50°C prior to use. Chlorophylls, primary and secondary carotenoids and α-tocopherol were identified and quantified with a RP C18 HPLC column protocol in combination with diode array and fluorescence detector in a row, according to Remias and Lütz (2007).

### Astaxanthin-ester comparison by liquid chromatography–mass spectrometry

The individual signatures of astaxanthin derivatives (peak size, retention time, molecular weight) of *Chlainomonas* sp*.* and *Cd.* cf. *nivalis* were compared with an Agilent 1100 HPLC system using a diode array detector, coupled with a Bruker Esquire 3000 mass spectrometer. Chromatographic conditions were as follows. HPLC column: Jupiter 5μ C18 300A, 250 × 2 mm (Phenomenex), thermostated at 25°C; solvent A: methanol; solvent B: acetonitrile–dichloromethane–methanol, 2:1:1; pumping rate: 0.4 mL min^−1^. Acetonitrile and methanol were of HPLC gradient quality. Linear mobile phase gradient: 1% B, 0–15 min; 1–77.7% B, 15–50 min; 100% B, 51–55 min (wash step); and 1% B, 55–65 min (reconditioning). Filters with freeze-dried algae were extracted in dichloromethane, and the supernatant was then vaporized and the residue resolved in methanol and centrifuged prior to injection of the supernatant. Mass spectrometry was performed using atmospheric pressure chemical ionization at positive mode (APCI^+^), drying temperature 250°C, ionization at 400°C, dry gas 10 L min^−1^ and vaporization at 50 p.s.i. Ion trap scanning was set from 200 to 1500 *m*/*z*. The MSMS mode was used for fragmentation of astaxanthin ester peaks to determine the presence of either a mono- or diester at a given retention time. Consequently, the liquid chromatography–mass spectrometry (LC-MS) set-up was used for the calculation of the native astaxanthin mono- to diester ratio by semi-quantitative summing of the integrated peak areas of both groups. Triglyceride peaks, which also occurred abundantly in the apolar extract and thus in the MS detector, were distinguished from astaxanthin esters with similar retention times by the missing spectral absorbance in the VIS region (chromatogram: 480 nm) and subsequently not used for the chemotaxonomic comparison. For the latter, the two largest MS ions of each astaxanthin ester peak were used in combination with their chromatographic retention time. Peaks with a height less than 50 mAU were not used due to MS signal noise.

## RESULTS

### Field observations

*Chlainomonas* sp. was sampled in the Austrian Alps during four growth seasons in a 7-year period. It was harvested from snow slush bedded on the surface ice cover of the high alpine Gossenkölle Lake, and one sample from waterlogged snow slush on glacial ice close to the Mount Dachstein (for location photographs, see Supplementary Figs 1 and 2). The exact geographic positions, elevations, pH (ranging from 5.4 to 5.9) and electrical conductivities (ranging from 2.6 to 7.4 μS cm^−1^) of snow meltwater are summarized in Table 1. The *Chlainomonas* population at Gossenkölle Lake was a regular local phenomenon, as it was observed every growing season sampled from 2008 to 2015, and harvested material consisted practically only of this species, despite the other snow algae *Cr.* cf. *nivalis*, *Cr. brevispina*, *Cr. rosae* var*. psychrophila* and *Cd.* cf. *nivalis* being present in the vicinity of the lake. For comparison, blooms of *Cd.* cf. *nivalis* were taken from seasonal snowfields above timberline. The population densities of *Chlainomonas* sp. were approximately 3 × 10^3^ (HW02) and 1 × 10^4^ cells mL^−1^ snow meltwater (GK12), compared with 5 × 10^4^ for *Cd.* cf. *nivalis* (HW16), resulting in a more intense appearance of the latter red snow.

### Cell structure and ultrastructure

**Figure 1.**
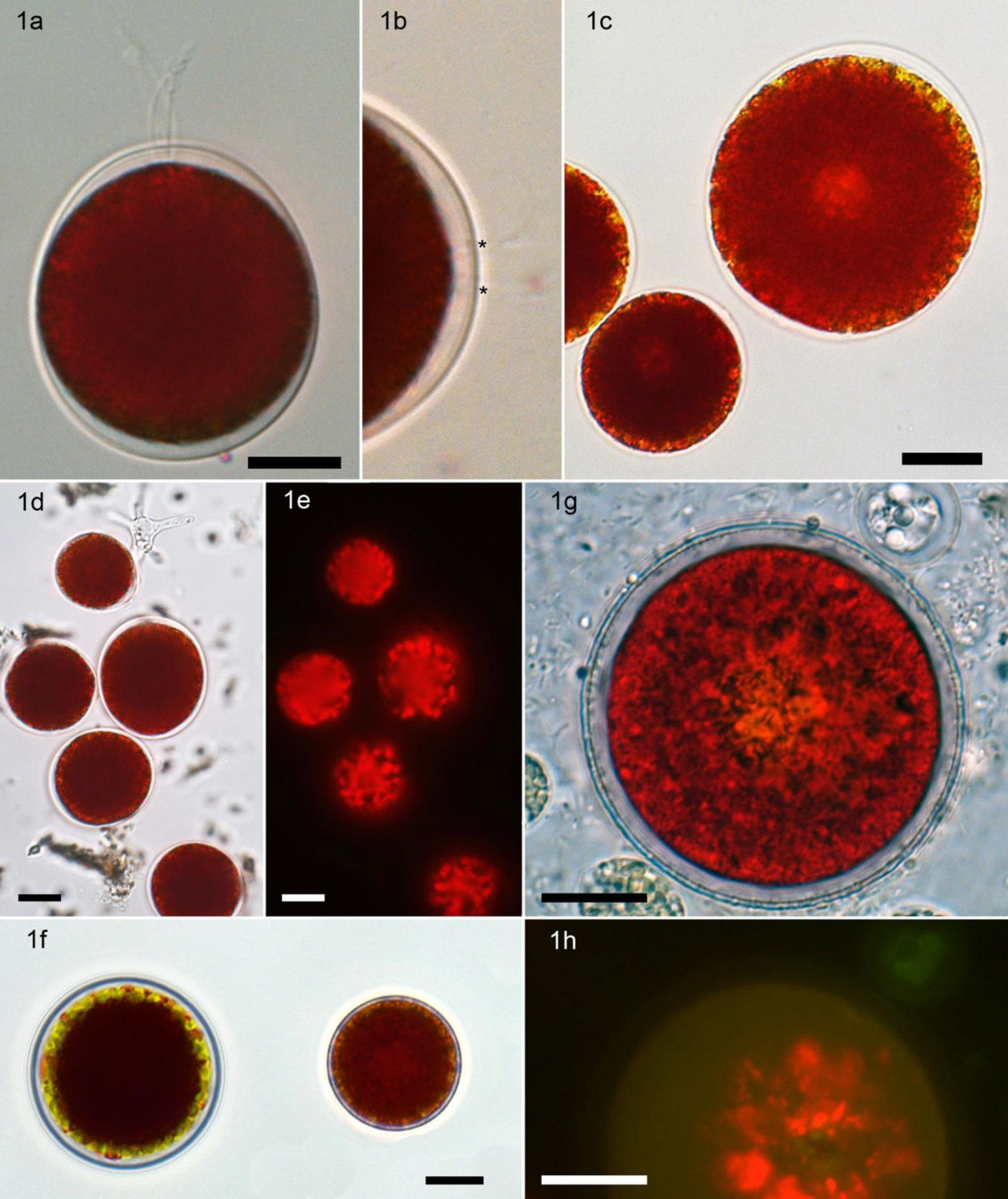
Light microscopy of *Chlainomonas* sp. field samples from snow at Gossenkölle Lake, Tyrol. (**a**) typical flagellate cell. (**b**) Detail of anterior cell wall apex, showing two pairs of flagella groves of a swarmer marked with*. (**c**) Non-motile cells of ellipsoidal shape and widened walls at the anterior and posterior cell poles. Note the brighter region in the centre of the big cell, representing the nucleus. (**d** and **e**) Group of cells in bright field (d) and chlorophyll-autofluorescence mode (e). Note the complex, irregular arrangement of parietal plastids. (**f**) Non-motile spores, spherical, the left one with a three-layered wall and a green plastidal ‘corona’ at the protoplast periphery. (**g** and **h**) Mature resting stage kept at laboratory conditions for 4 months. The innermost cell wall became thicker and less translucent (g), and the fluorescence mode (h) showed that plastids are arranged more centrally. Bar: 10 μm.

*Chlainomonas* sp. had swarmers with four flagella and their length was about the same as that of the cells (Fig. 1a). They were seen moving slowly when observed by LM and without notable cell rotation. Flagella were oriented at the backside during the movement (see Supplementary Video 1). They retained functionality only when observed below 2°C, and otherwise they were discharged within seconds when cells were exposed to higher temperatures. Occasionally cells had less than four flagella. The motile cells were of ellipsoidal shape with average sizes of 26–36 μm in length and 23–33 μm in width (see Table 1). A typical papilla was usually not present, but four flagella grooves were observed in the cell envelope (Fig. 1b). A very characteristic detail of this stage was, to a varying extent, widened and partially thickened smooth cell walls in the anterior and posterior section (Fig. 1c and d). The protoplast was more or less circular; further details were almost entirely obscured by the red secondary pigments, but with three exceptions. First, four contractile vacuoles were present at the anterior apex; second, a central more or less spherical region with a diameter of about 2–3 μm appeared brightened in LM, representing the location of the nucleus (Fig. 1c), as proved by TEM (see below); and third, the lipid bodies containing astaxanthin frequently did not reach the margins of the protoplast, but instead many small plastids (position and morphology made visible using chlorophyll auto fluorescence; Fig. 1e) were located parietally and sometimes visible as a more or less greenish ‘corona’ (Fig. 1f). These three characteristics were never observed for the morphologically similar *Cd.* cf. *nivalis*.

The successional cell stage was a mature ‘resting spore’. These either occurred in samples collected later in the season just before complete melt of the lake ice, or developed from swarmers kept in meltwater at 4°C in the laboratory for several weeks (Fig. 1g). These cells lost flagella, had no partial thickened outermost cell wall anymore and were always spherical. Alternatively, the chloroplasts were located around the central nucleus as indicated by their auto-fluorescence (Fig. 1h). A new, characteristic secondary cell wall was formed inside, and it became thicker and less translucent during aging, maybe accompanied by incrustation. The outer cell wall remained thin, was not incrusted and had a dark outer margin. A third or multiple thin outermost and transparent envelopes (Fig. 1g) frequently accompanied it. Further cellular details like stigma, spines on the wall exterior, cell divisions or any other stages in the life cycle were not observed in field material. Occasionally, some small red flagellates with prominent papilla but uncertain taxonomic affiliation were found (Supplementary Fig. 3).

**Figure 2.**
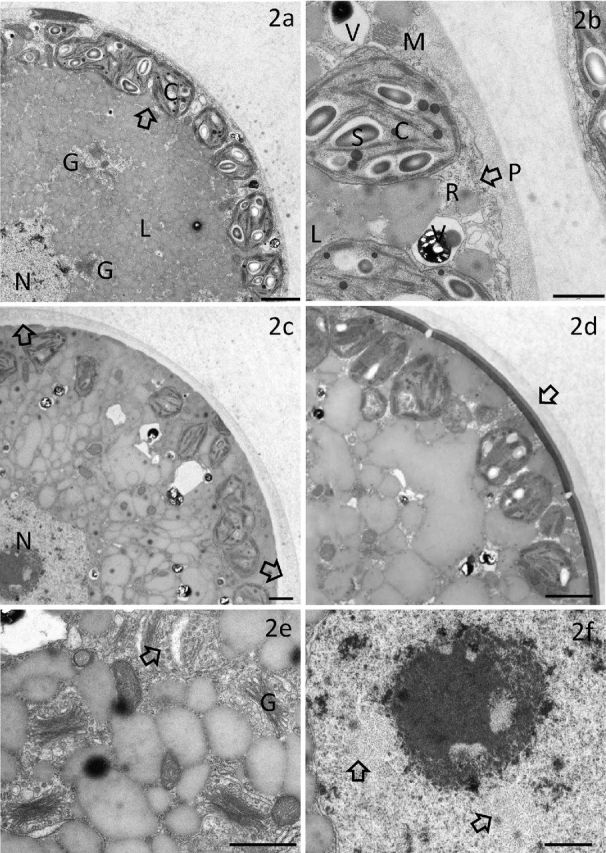
Transmission electron microscopy of *Chlainomonas* sp. (**a**) Single-walled cell with a peripheral arrangement of many small plastids (C); note a possible process of division (arrow); numerous lipid bodies (L) encircle the central nucleus (N). (**b**) Detailed view of a plastid (C) with starch grains (S) and thylakoid grana; the cytoplasm also contains mitochondria (M), ribosome-rich regions close to the cell wall (R), undulated cell membrane (P) and vacuoles (V) of unknown function with electron-dense content, which is partly crystallized. (**c**) Cell stage with bi-layered wall with species-typical widened outer wall at the ant- and posterior (arrows). (**d**) Mature spore with three-layered cell wall (arrow); the innermost is very electron dense. (**e**) Highly active Golgi cisternae, one of them in cross sectional view (arrow). (**f**) Detailed view of nucleus (N) with central nucleolus and putative DNA cluster arrangements (arrows). Bar: 1 μm.

More detailed information on *Chlainomonas*’ cytoarchitecture was gained using TEM. The nucleus was centrally located and surrounded by numerous lipid bodies (Fig. 2a). Typical was a peripheral arrangement of many small plastids containing starch grains but no pyrenoid. Of note were several vacuoles of unknown function with electron-dense content, which was partly crystallized (Fig. 2b). Figure 2c shows a cell with bilayered cell wall; the outer cell wall layer was widened at the anterior and posterior typical of swarmers of this species. Mature spores with three cell wall layers (Fig. 2d) had plastids not directly located below the cell membrane, and one can assume a high physiological activity through the presence of many Golgi bodies with attached vesicles (Fig. 2e). The nucleoplasm contained occasional remarkable strand-like clusters (Fig. 2f). In contrast, TEM of *Cd.* cf. *nivalis* (Supplementary Fig. 4) showed only single-walled spores of spherical shape and attached cryoconite particles. There was always one central chloroplast with naked pyrenoid, surrounded by lipid bodies.

### Phylogenetic analyses

**Figure 3.**
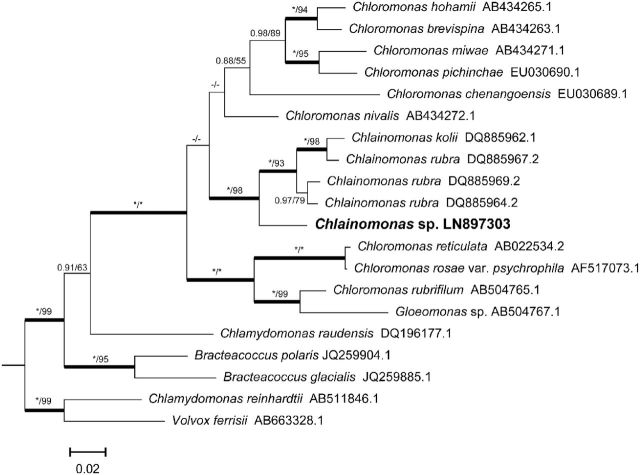
Bayesian tree using *rbc*L, including Chlamydomonadalean algae isolated from either snow or polar habitats (except mesophilic outgroup at the bottom). *Chlainomonas* sp. from the Tyrolean Alps (sample DR67, bold) groups with the two other snow algae of this genus, *Cn. rubra* and *Cn. kolii* from North America and New Zealand, respectively*.* The genus *Chlainomonas* itself is part of the ‘*Chloromonas* snow’ clade shown in Remias *et al*. (2010). Values at the branches indicate Bayesian posterior probabilities (PP) and maximum likelihood bootstrap (BS) values. Asterisks indicate BI PP = 1.00 and ML BS = 100; dashes indicate BI PP < 0.8, and ML BS < 50.

Phylogenetic analyses of the *rbc*L sequence placed the field sample *Chlainomonas* sp. DR67 among other species of the genus *Chlainomonas*. All species of the genus form a well-supported monophylletic clade (Fig. 3). Interestingly, *Cn. rubra* and *Cn. kolii* from North America and New Zealand were more closely related to each other than to *Chlainomonas* sp. from the Austrian Alps. Consequently, no species affiliation was performed for the samples of this work. Other close relatives of *Chlainomonas* were several species of *Chloromonas* that also live in snow.

### Photosynthesis

**Figure 4.**
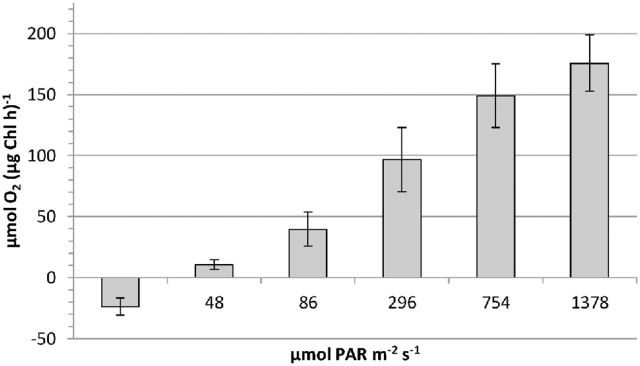
Oxygen turnover during dark respiration (first bar) and light-dependent photosynthesis (five bars with irradiation value stated on the *x*-axis) of *Chlainomonas* sp. (sample GK04) at 1°C, normalized to the amount of chlorophyll per sample. PAR, photosynthetic active radiance.

Dark respiration and light-dependent oxygen production were measured at 1°C, close to ambient conditions. The photosynthetic performance was tested under five different light levels (Fig. 4). At low light conditions (48 μmol PAR m^−2^ s^−1^) a net oxygen production of 10.6 ± 3.9 μmol O_2_ (mg Chl h)^−1^ was already achieved, considering a more than two-fold oxygen consumption during the dark phase. No photoinhibition was observed up to 1378 μmol PAR m^−2^ s^−1^.

### Pigments

HPLC of apolar extracts revealed astaxanthin as the only secondary carotenoid of *Chlainomonas* sp. It causes the red coloration of cells due to its abundance compared with chlorophylls and primary carotenoids. Cells have about five times more astaxanthin than chlorophyll a (sample AS02). Further pigment contents (relative ratios of pigments and of α-tocopherol to chlorophyll a in comparison with *Cd.* cf. *nivalis*) are given in Supplementary Table 2. A representative chromatogram of *Chlainomonas* sp. (Supplementary Fig. 5) shows a large number of peaks with varying size and identical online absorption maxima either at 470 nm, or alternatively at 466 nm in combination with an additional side maximum at 370 nm. Their spectral absorptions are in accordance with all-*trans* and 13 *cis*-astaxanthin standards (Supplementary Fig. 6), which, however, had much earlier chromatographic retention times of 2.2 and 2.5 min than the algal peaks (from 3.7 to 45.1 min). Consequently, astaxanthin was accumulated natively for the most part as a number of different derivatives (e.g. more than 99% of total astaxanthin peak area in sample AS02), the secondary carotenoid therefore becoming more apolar.

Despite the same red cell color, HPLC of *Cd.* cf. *nivalis* interestingly exhibited many differences in relative peak sizes and retention times of astaxanthin derivatives (Supplementary Fig. 7). For instance, very apolar peaks with high retention times were relatively small, while *Chlainomonas* sp. typically had relatively larger peaks in this region (Supplementary Fig. 5). These characteristic patterns of the two species were constant, regardless of collecting season or growing site in the Austrian Alps. Additionally, a sample of *Cn. rubra* from New Zealand (courtesy of Phil Novis, Landcare Research, Lincoln, NZ) exhibited a chromatogram like the populations of this study from Europe with a characteristic dominance of peaks with late retention times and α-tocopherol levels much higher than in *Cd.* cf. *nivalis*.

### Comparison of astaxanthin derivatives

Taking the species-specific constancy of astaxanthin derivatives in chromatograms of both snow algae into account, independent of the collection year and location, a tentative comparison was undertaken, using the two main parameters of molecular size (*m*/*z*) and compound polarity (retention time). Additionally, the abundance of pigment derivative classes was calculated. Therefore, LC-MS was applied with apolar extracts to gain information about the nature of astaxanthin derivatives in detail. Using APCI^+^, the detector received carotenoid ions as [M+H]^+^. This, and the mode of astaxanthin fragmentation during MS, was clarified by injecting two astaxanthin standards (all-*trans* and 13-*cis* isomer). A characteristic phenomenon was an *m*/*z* = –18 split-off and a minor *m*/*z* = –36 split-off from the native compound with the all-*trans* and 13-*cis* isomer, respectively (Supplementary Fig. 8). Most likely one water [M+H-18]^+^ and two water molecules [M+H-2×18]^+^, respectively, were cleaved from the carotenoid. The same mechanism was found frequently for astaxanthin derivatives, either spontaneously without active MS fragmentation or during the active MSMS fragmentation process, too. By fragmentation, astaxanthin mono- and diesters were identified by cleavage of either one or two fragments, respectively, with a size typical for algal fatty acids, and an astaxanthin residue was finally left. A representative example of diester fragmentation is given in Supplementary Fig. 9.

**Figure 5.**
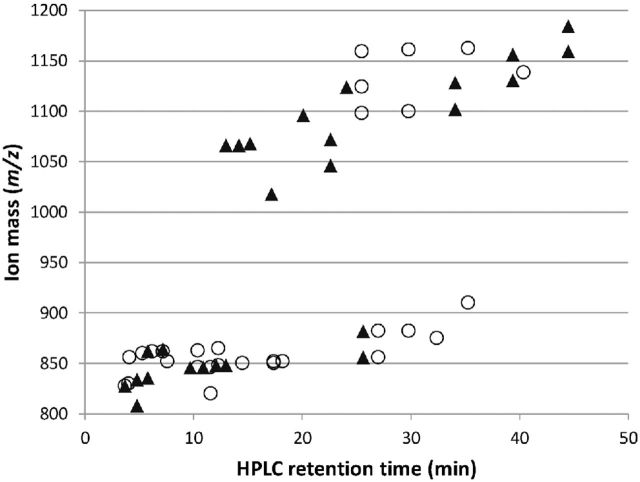
Comparison of molecular sizes (*x*-axis) and polarities (*y*-axis) of astaxanthin esters from *Chlainomonas* sp. (AS02; open circles) and *Cd.* cf. *nivalis* (DR48; filled triangles), acquired by LC-MS. The two strongest MS^+^ ions of each peak occurring at a given HPLC retention time were included. Higher retention means less polarity. The ion mass (*m*/*z*) represents the molecular size of the compounds. Using the information of MSMS fragmentation patterns, two size classes became distinguishable, namely from about 800 to 925 comprising monoesters, and from about 1025 to 1175 comprising diesters. Both snow algae show some similarities at lower retention times (monoester section) and more differences are present for higher retention times (diester section).

The abundances of astaxanthin derivatives were compared between *Chlainomonas* sp. and *Cd.* cf. *nivalis* (Fig. 5)*.* In each species, astaxanthin was esterified with one or two fatty acids of different size and polarity. Both snow algae had similar pigment monoesters in terms of size and polarity (at lower retention times), but a more individual pattern was present for higher retention times and for diesters in general. In a final step, the ratios of mono- to diesters of astaxanthin of both species were compared by summing the respective HPLC peak areas (Supplementary Table 3). As a prominent difference, *Chlainomonas* sp. stored the majority of astaxanthin derivatives in the diester form, and to the contrary, *Cd.* cf. *nivalis* stored them in the less apolar monoester form. There were no indications of any further kind of astaxanthin derivatives in any of the samples from both species.

## DISCUSSION

This study was performed with field material with the intention of describing *Chlainomonas* sp. in its authentic physiological and cytological state in alpine habitat conditions. Generally, chloromonads in culture exhibit a very different phenotype, without secondary pigments, hardly forming any resting stages, or with striking cytoarchitecture such as secondary wall structures or chloroplast shape rearrangements. Moreover, many snow algae are not available in culture collections, though one field sample collected for this study (HW02) was deposited in the CCCryo collection (Fraunhofer, Potsdam-Golm, Germany) and transformed into a green strain by cultivation (strain CCCryo 345-09 ‘*Chloromonas* sp.’).

The slightly acidic pH values and low electrical conductivities of algal snow meltwater were similar to those for *Cd.* cf*. nivalis* and many other snow algae of habitats with low nutrients (Hoham and Duval 2001; Hoham *et al.*^2007^). Spijkerman *et al.* (2012) suggested that the distribution of snow algae also depends on topographical and geological parameters like slope, occurrence of meltwater rivulets and rock formation.

Only a few reports of *Chlainomonas* red snow (in contrast to *Cd. nivalis*) exist, for which there are a number of reasons. One factor is the special ecological requirements. The preferred habitat of *Chlainomonas* sp. used in this study is waterlogged snow, which is mainly the case at ice covers of high mountain lakes and in snow bedded on plain glacier surfaces. Novis (2002b) found that growth of *Cn. kolii* was boosted after heavy rainfall during alpine summer, causing wetter snow conditions. Probably these spores are, despite possessing multiple cell wall layers compared with *Cd. nivalis*, more sensitive to both desiccation stress and higher temperatures, which would be the fate in non-permanent and rock-based snowfields after complete melt every season. In fact, at the Gossenkölle Lake *Chlainomonas* sp. ends up in water after complete melt, and thus protected from higher temperatures and desiccation; however, the complete life cycle including spring time with cell divisions and putative migration to the ice surface remains to be investigated. In summer, as the cells lose flagella after complete snow melt, they will most likely sink to the lake bed, or in the case of the samples from the Hallstätter glacier, remain on an icy surface. To the contrary, *Cd.* cf. *nivalis* seems to be adapted to temporary warmer conditions (Lukeš *et al*. (2014) consider it to be a ‘cryotolerant mesophile’), because cells were found intact on soil or rock surface after snow melt, whereas no populations from slushy sites were ever reported. Also *Cn. rubra* and *Cn. kolii* differ in their habitat from *Chlainomonas* sp., as they were found mostly associated with coniferous tree canopies, where snow was not slushy but shaded much of the day (Hoham 1974a,b).

A significant diagnostic detail valid for the LM description of *Chlainomonas* is a cell wall throughout ‘distant’ from the protoplast (Ettl 1983). However, *Chlainomonas* sp. from the Austrian Alps only possesses partially thickened walls practically at the anterior and posterior cell poles (representing a kind of papilla and pseudopapilla). The populations were morphologically identical neither to the descriptions of *Cn. kolii* (swarmers with collar-like papilla, spores with outer, ephemeral envelope consisting of mosaic plates) nor to *Cn. rubra* (cell wall of swarmers thickened all around, spores with striking spikes at the surface). However, similarities were found to other poorly described taxa known from red snow, namely *Cr. bolyaiana* (which, however, is much larger than *Chlainomonas* sp.) and *Cd. sanguinea* (about the same cell sizes and thickened cell walls at the poles; however, flagella 1.5–2 times cell length and spores with only two wall layers). The fact that cell shape and plastid morphology (respectively their arrangement) of Chloromonadalean algae can change drastically depending on the stage of the life cycle has already been shown for *Cr. nivalis* (Remias *et al.*^2010^), and it is most likely the same for *Chlainomonas* sp. For example, the numerous small plastids of spores can be rearranged from a peripheral to a central position close to the nucleus. Moreover, they probably originate from a larger single chloroplast in an earlier stage of the life cycle. In general, environmental factors and culture conditions may influence details like the presence or peculiarity of a pyrenoid or stigma, which were classically regarded as important diagnostic details. Summarizing, species determination using LM remains difficult, as cell shape and chloroplast morphology may change during development, and earlier descriptions like those summarized in Kol (1968) were performed on fixed material and without any life cycle studies. Consequently, older reports of *Cr. bolyaiana* and *Cd. sanguinea*, which look very similar to *Chlainomonas* ssp., have to be scrutinized. Still, a separation of *Chlainomonas* ssp. against *Cd. nivalis* field samples at the LM level should be possible, taking into account that the first species is usually larger (average cell lengths clearly more than 25 μm) and possess either ovoid stages with (partly) thickened cell walls with four flagella (groves) or spherical spores with multi-layered cell walls. Moreover, mature spores are not covered with cryoconite. Considering these details, a study of Kawecka (1981) probably did not describe spore formation of *Cd. nivalis*, but instead of a *Chlainomonas* living in the Polish High Tatra Mountains.

TEM was performed because large amounts of astaxanthin mask taxonomically relevant details at the LM level. It was shown that the cell walls of *Chlainomonas* sp. were not ‘detached’ from the cytoplasm as initially described for this genus and that is typical for the green alga *Haematococcus pluvialis* (Ettl 1983)*.* Instead, the outermost wall layer is usually thickened and consists of less electron dense cell wall material. This may also be the situation for certain stages of *Cn. kolii* or *Cn. rubra*. The innermost cell wall layer of mature, multi-layered spores with its higher electron density may play a role in long-term survival. Novis (2002b) showed by TEM that *Cn. kolii* possesses small peripheral plastids (like the species from the Austrian Alps), and this phenomenon seems to be typical within the *Chloromonas* ‘snow clade’, as shown for other species, e.g. *Cr. polyptera* (Remias *et al*. 2013) or *Cr. chenangoenis* (Hoham *et al.*^2006^; Matsuzaki, Hara and Nozaki 2014). The small pyrenoid-free plastids may also be arranged inside the cell surrounding the nucleus. It remains open whether these small plastids are independent or in practice only portions of a single chloroplast. A physiological benefit of this strategy could be a better metabolite exchange with the cytoplasm at low temperatures due to a better membrane surface to volume ratio. The existence of many small cytoplasmic vacuoles with electron dense content that is partly crystallized was described earlier for *Cd.* cf. *nivalis* (Lütz-Meindl and Lütz 2006), but their physiological role remains unknown. They seem to be common in green algae, but their abundance in some snow algae is striking; e.g. in the recently described *Cr. krienitzii* from Japan (Matsuzaki *et al.*^2015^), they were visible by means of LM and TEM. A further characteristic detail of *Chlainomonas* ssp. and *Chloromonas* ssp. snow algae is the strictly centrally located nucleus, which is sheltered from excessive high alpine UV radiation by the surrounding astaxanthin. In contrast, *Cd.* cf. *nivalis* has no prominent central nucleus, but a single axial chloroplast with naked pyrenoid instead (Supplementary Fig. 8). Moreover, the cell walls are single-layered, typically possess attached cryoconite particles and are never partially thickened. Finally, spores of this species are generally smaller (around 20 μm in diameter). In summary, despite similarities of mature, spherical spores of *Chlainomonas* sp. and *Cd. nivalis*, significant differences in structural details exist.

Initially, snow algae of the genus *Chlainomonas* were part of *Sphaerellopsis* (Kol 1968), a diagnosis based on cells with only two flagella. However, already Hoham (1974a) described practically all cells as possessing four flagella grooves in the cell wall, regardless of individual swarmers (which may lose some or all of the flagella, e.g. when the cells are stressed during LM), and consequently transferred them to *Chlainomonas*. The quadriflagellate nature of *Cn. rubra* was later confirmed by Novis *et al.* (2008) by TEM. The same authors showed the first molecular tree of snow algae including *Cn. rubra* and *Cn. kolii* by using *rbc*L sequences. Surprisingly, they found that the phylogenetic position of *Chlainomonas* was within the genus *Chloromona*s s.l., with *Cr. chenangoensis* from snow in northwestern America (Hoham *et al*. 2006) as the closest known relative. So far, all known species of *Chloromonas*, which are closely related to *Chlainomonas*, inhabit snow fields, which points to a common ancestor of this cryoflora lineage. However, these *Chloromonas* spp. from snow have a quite different morphology*.* Spores in the *Chloromonas* spp. clade typically possess small discoid plastids. *Chloromonas* forms overall smaller spores of fusiform shape when compared with *Chlainomonas*, and the cell walls are covered with prominent protuberances like flanges and spikes. Spiked cell walls were reported for a stage of *Cn. rubra* (Hoham 1974a), but we never found such a cell in alpine samples. Finally, the secondary pigmentation of *Chloromonas* spores from snow is less developed; they never accumulate astaxanthin to such an extent as *Chlainomonas* so as to be confused with *Cd.* cf. *nivalis*. Neither molecular data nor strains are available for *Cr. bolyaiana* and *Cd. sanguinea*, which could, due to similar physiology and morphology, possibly be located close to the genus *Chlainomonas*. 18S rDNA sequences of *Chlainomonas* sp. from Gossenkölle Lake were already previously registered at GenBank (entries GU117574 and GU117575, sample code HW01, designated as *Chloromonas* sp.). *Chlamydomonas* cf. *nivalis* from the Austrian Alps (Tyrol) was included as GU117577 (sample code P27/DR1). Remias *et al.* (2010) demonstrated using 18S rDNA that the alpine *Chlainomonas* sp. was embedded within the ‘*Chloromonas* snow’ clade, and thus the results are in accordance to those from *rbc*L analysis (see above). In contrast *Cd.* cf. *nivalis* from the Tyrolean Alps was shown to group with different members of the Chlamydomonadaceae forming the ‘red snow’ clade. Most recently, in an 18S phylogeny of Matsuzaki *et al*. (2015, fig. S13), two strains related to *Chloromonas* species and isolated from arctic Svalbard were recognized as the closest relatives of alpine *Chlainomonas* sp. Future investigations on biodiversity of polar and alpine snow and ice ecosystems should include a new approach presented by Lutz *et al*. (2015): using high-throughput sequencing of the small subunit ribosomal RNA gene with environmental samples taken from glaciers in Iceland, they found an overwhelming diversity of bacteria, archaea, fungi and algae, and could also roughly measure the abundances of these organisms on snow and ice.

Photosynthesis of snow algae has to cope with different kinds of abiotic stresses. Quite different light conditions can occur. On the one hand, almost darkness prevails when cells are situated deep below the surface of snow at the onset of summer. On the other hand, exposed cells at unshaded snow surfaces can be subject to very high VIS und UV irradiation. Membrane flexibility and protein functionality have to adjust to these changing conditions. Lukeš *et al*. (2014) were the first to elucidate cellular adaptations of *Cd.* cf. *nivalis*: they described thylakoid membranes that were mainly composed of negatively charged phosphatidylglycerol. Also different from conventional green algae, the photosynthetic D1 protein had increased structural flexibility owing to amino acid changes. This in part explains why this species is successful in non-permanent snow fields, where cells are exposed to significantly higher temperatures when on bare soil or rock after complete melt. Whether such adaptations exist for *Chlainomonas* sp. is unknown. At low temperatures, this species has about the same maximum photosynthetic performance values of approximately 175 μmol O_2_ μg Chl^−1^ h^−1^ as other snow algae such as *Cr. nivalis* (Remias *et al.*^2010^) and *Cd. cf. nivalis* (Remias, Lütz-Meindl and Lütz 2005).

So far, astaxanthin has proven to be the secondary pigment turning Chlamydomonadalean snow algae red, and this has been shown here for the first time for *Chlainomonas*. The two hydroxyl groups of this carotenoid allow a simple chemical modification via esterification with any fatty acid. Chain length of fatty acids and the presence or number of double bonds allows the alga to optimize the polarity of astaxanthin in accordance with storage conditions in their lipid bodies, which is influenced by the nature of the triglycerides present. Řezanka *et al.* (2014) performed lipidomic profiling with the snow alga *Cr. pichinchae* using high resolution LC-MS, and were able to characterize the triglycerides. They found triglyceride species from 0 to 12 double bonds and they contained only C16 and C18 fatty acids with 0 to 4 double bonds, indicating a high presence of poly-unsaturated fatty acids, which are beneficial in cold environments as they enhance the membrane fluidity. In an earlier study, Řezanka *et al.* (2013) characterized a new variation on astaxanthin derivatives, namely esters with glucose and fatty acids. Although they analysed a field sample of *Cd.* cf. *nivalis* collected close to our Tyrolean study site, we could not detect such glycolized astaxanthin in our samples by LC-MS. Glycolized astaxanthin is recognized by a characteristic MSMS *O*-glycosidic fragmentation of the monosaccharide from the carotenoid.

*Chlainomonas* sp. from the Alps and a sample of *Cn. kolii* from New Zealand (and consequently maybe the whole genus) had exceptional variants of astaxanthin: the majority was stored as di- instead of monoesters and were generally more apolar than those of *Cn.* cf. *nivalis.* This goes along with high levels of α-tocopherol, a major antioxidant of the chloroplast, which can probably compensate damage from high light stress (excessive VIS) to exposed plastids anchored directly below the cell wall.

This study has shown that spores of snow algae can evolve quite different cellular strategies to adapt to the special snow environment. The photosynthesis of *Chlainomonas* sp. shows good performance under ambient light and temperature conditions, so that the cells can accumulate reserve metabolites like starch and astaxanthin esters for overwintering. Although the complete life cycle of this species is unknown, it seems to prefer summer snow with high water content in areas where cells are not subject to desiccation or high temperatures after snow melt. Consequently, we found this organism at locations where the flat snow fields had low water drain during melting. The psychrophilic nature of *Chlainomonas* sp. was not tested, but the fact that swarmers lose their flagella when exposed to temperatures above 1°C points to this direction. Similar observations on dropping flagella were made by Hoham (1974a). The high abundance of very apolar astaxanthin diesters differentiates *Chlainomonas* in its chemotaxonomic profile from the other two main groups of Chlamydomonadalean snow algae, the ‘*Chloromonas*-snow’ clade and the ‘*Chlamydomonas-*red snow’ clade.

## Supplementary Material

Supplementary Data
