# Supplementary material for: Ecophysiology, secondary pigments and ultrastructure of Chlainomonas sp. (Chlorophyta) from the European Alps compared with Chlamydomonas nivalis forming red snow
Source: FEMS Microbiol Ecol. 2016 Feb 15;92(4):fiw030. doi: 10.1093/femsec/fiw030 (PMC4815433; doi:10.1093/femsec/fiw030)
Supplement: Supplementary Data [file fiw030_supplementary_data.zip › Supplement_final_R1.docx]

**Ecophysiology, secondary pigments and ultrastructure of *Chlainomonas* sp. (Chlorophyta) from the European Alps compared to *Chlamydomonas* *nivalis* forming red snow**

**Supplementary Data**

| sample | date | location | GPS | altitude m | pH | el. cond. | diameter |
| --- | --- | --- | --- | --- | --- | --- | --- |
|  |  |  |  |  |  | *µ*S cm^-1^ | *µ*m |
| HW16 | 11 Sep 2008 | Tiefenbach glacier | N46°55.009 E10°55.914 | 2975 | 4.6 | 2.9 | 20 ± 1.5 |
| DR23 | 19 Aug 2004 | Kühtai | N47°13.345 E11°01.000 | 2300 | 5.8 | 10.0 | 20.4 ± 2.9 |
| DR36 | 04 Jul 2006 | Kühtai | N 47°12' N E11°02' | 2450 | 5.7 | 1.9 | 19.6 ± 3.7 |
| DR48 | 26 May 2009 | Kühtai | N47°13.345 E11°01.335 | 2293 | 4.7 | 4.2 | nd |
| GK01 | 02 Jun 2009 | Kühtai | N47°13.738 E11°00.922 | 2410 | nd | nd | nd |
| DR52 | 10 Jul 2009 | Ötztal, Rotmoos | N46°50.270 E11°02.129 | 2309 | 4.9 | 4.5 | 26.7 ± 4.8 |

**Supplementary Table 1.** Samples of red snow caused by *Chlamydomonas* cf. *nivalis* with date of collection, name of location in Tyrol (Austrian Alps), geographic position and altitude. Furthermore, pH and electrical conductivity of snow meltwater, as well as average cell sizes are given, if available. Abbreviations: el. cond., electrical conductivity; nd … not detected/not available

|  | neo | vio | ast | asi | ant | lut | zea | chl b | β-car | ast-e | ast-tot | asi-e | asi- tot | α-toc |
| --- | --- | --- | --- | --- | --- | --- | --- | --- | --- | --- | --- | --- | --- | --- |
| *Chlainomonas* sp. | 0.067 | 0.052 | 0.045 | 0.000 | 0.023 | 0.189 | 0.039 | 0.489 | n.d. | 3.752 | 3.799 | 1.479 | 1.479 | 0.100 |
| *Cd.* cf. *nivalis* | 0.045 | 0.048 | 0.049 | 0.046 | nd | 0.106 | nd | 0.334 | 0.016 | 6.305 | 6.356 | 2.139 | 2.188 | 0.006 |

**Supplementary Table 2.** Relative content of pigments and α-tocopherol in ratios to chlorophyll a (=1) in field samples of *Chlainomonas* sp. (sample AS02) and *Cd.* cf. *nivalis* (sample DR36), determined by HPLC. Note that in *Chlainomonas* sp. the de-epoxydised xanthophyll-cycle pigments (ant, zea) are present, and it has also a much higher content of α-tocopherol. The total amount of astaxanthin (ast+asi and esters thereof) is 8.544 to 5.278 in favour to *Cd.* cf*. nivalis*. Abbreviations: neo, neoxanthin; vio, violaxanthin; ast, astaxanthin (*all*-trans); asi, astaxanthin *cis*-isomers (mainly 13*Z*); ant, antheraxanthin; zea, zeaxanthin; chl b, chloropyll b; β-car, β-carotene; ast-e, astaxanthin esters; ast-tot = ast+ast-e; asi-tot, total amount of astaxanthin *cis*-isomers; α-toc, α-tocopherol; n.d., not detected.

| sample | monoesters % | diesters % |
| --- | --- | --- |
| DR23 | 90.4 | 9.6 |
| DR47 | 91.2 | 8.8 |
| DR52 | 95.3 | 4.7 |
| DR53 | 31.7 | 68.3 |
| AS02 | 18.7 | 81.3 |

**Supplementary Table 3.** Percentages of astaxanthin mono- and diesters in field samples of *Chlainomonas* sp. ( DR53, AS02) compared to those of *Cd. nivalis* (DR23, DR47, DR52), calculated from according summed chromatogram peak areas at 480 nm and identified via LC-MS. Free astaxanthin, which was rare in both species, was not taken into account. *Cd. nivalis* has generally a larger relative content of monoesters compared to *Chlainomonas* sp., and the situation is vice-versa with diesters.

**Supplementary Figure 1.** Melting ice cover of the high alpine Gossenkölle Lake (Tyrol, Austria) with pink slush containing *Chlainomonas sp.* (Sample DR67, June 2015). Inset: harvest of algae at the lake (June 2009).


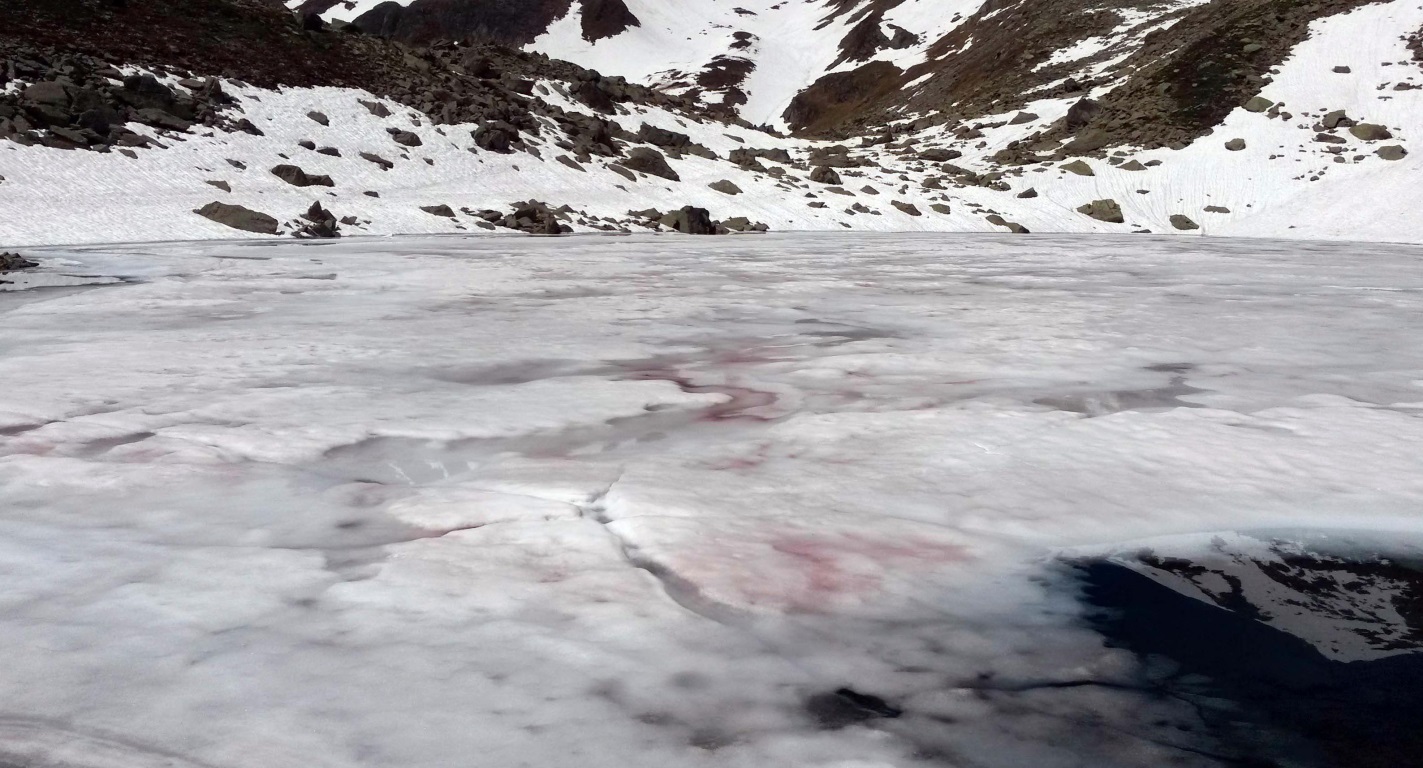

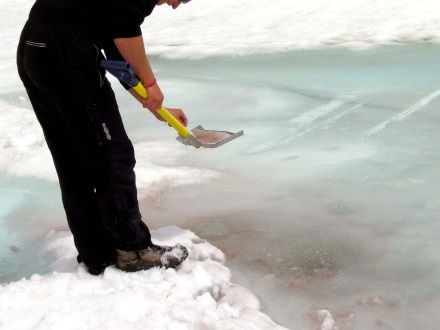

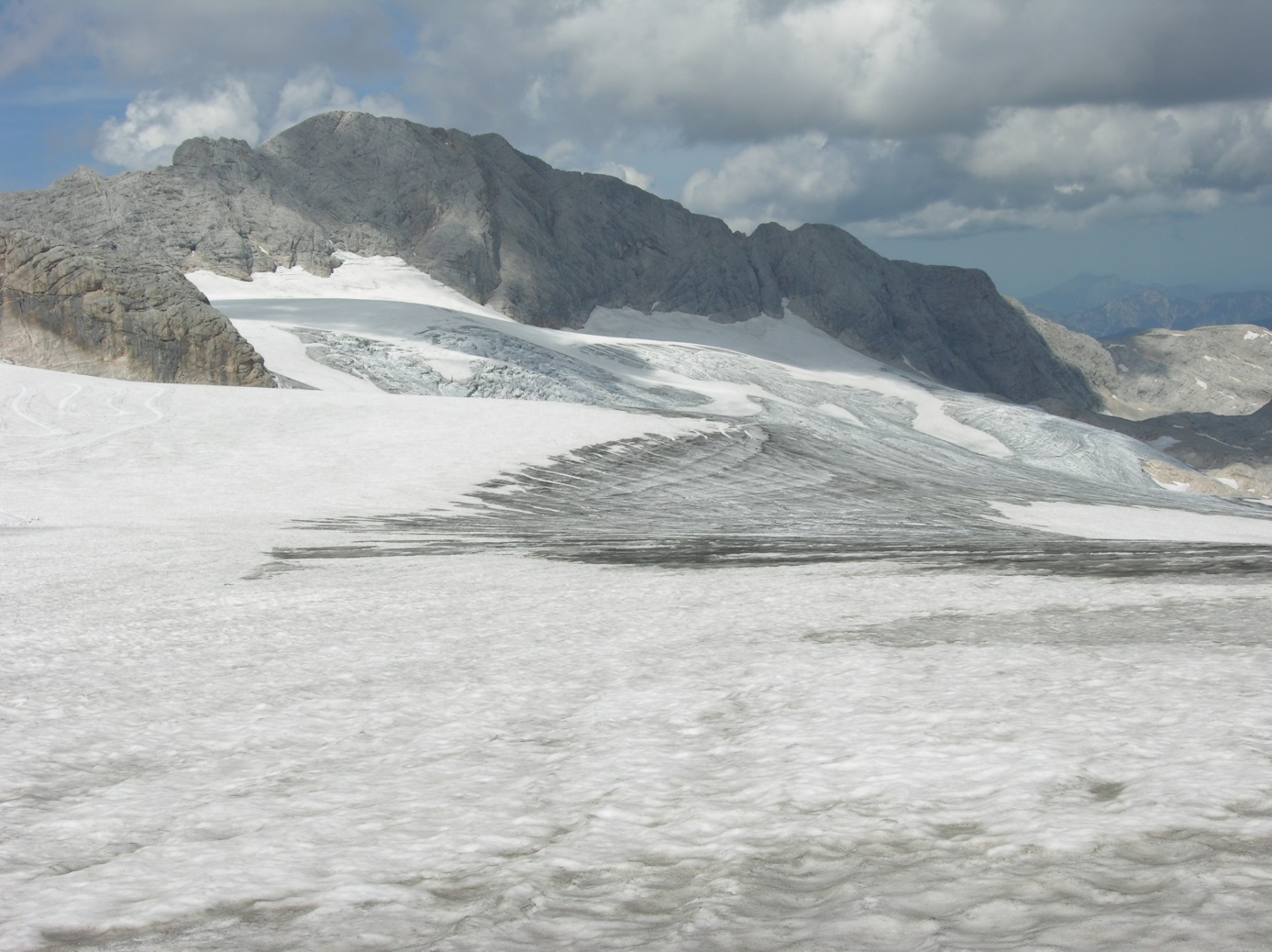

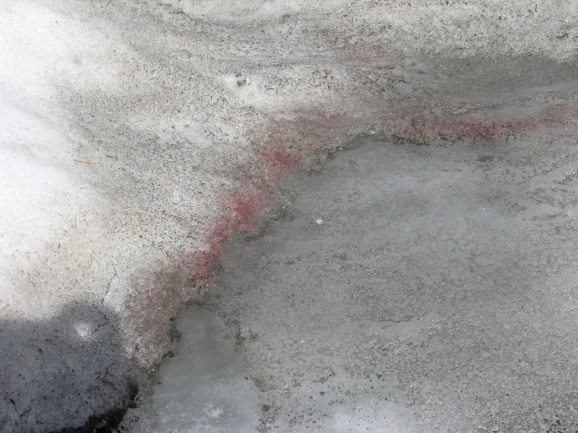


**Supplementary Figure 2.** Hallstätter glacier (Upper Austria) partly covered with snow and open areas of dark ice with cryoconite (July 2010). The limestone Dachstein massif is in the background (highest peak ‘Hohes Kreuz’ to the left, 2837 m a.s.l.). Inset: Snow field margin bedded on ice, coloured red by *Chlainomonas* sp (Sample DR53).


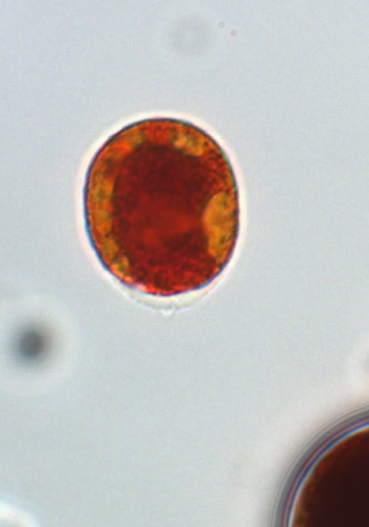

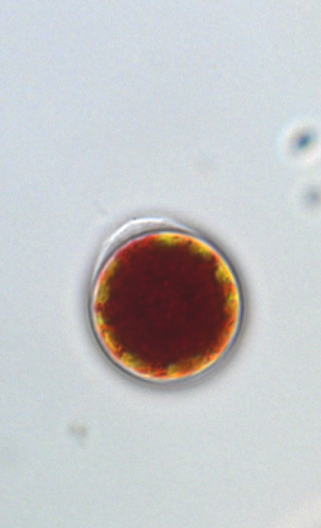

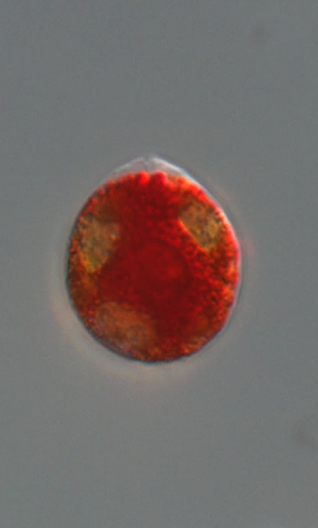

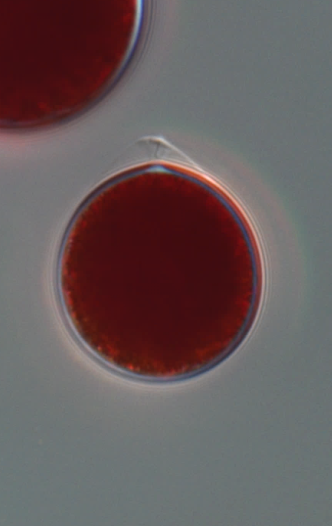


**Supplementary Figure 3.** LM of red flagellates or flagellate-like cells with papilla, which were rarely found in field samples consisting *Chlainomonas* sp. (Sample AS01) at Gossenkölle Lake, however with uncertain taxonomic affiliation. Bar: 10 *µ*m.


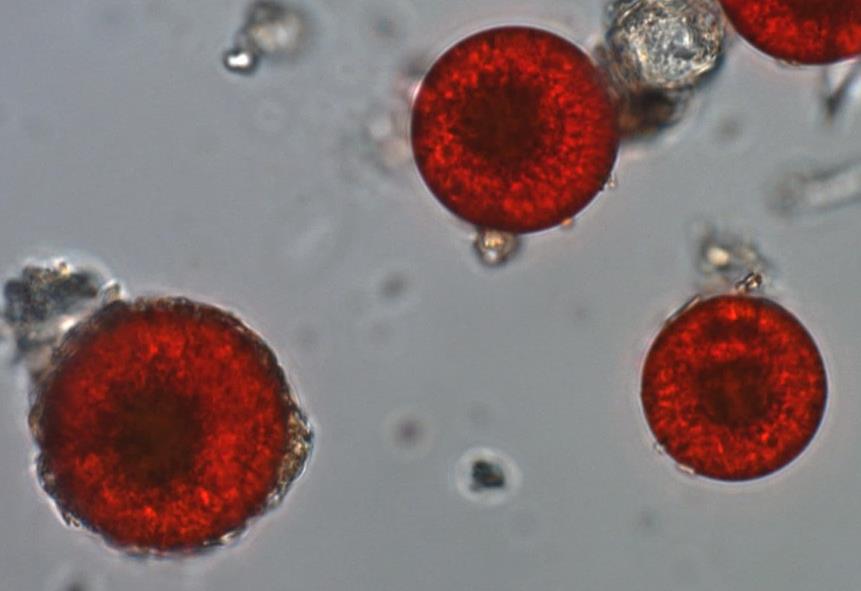

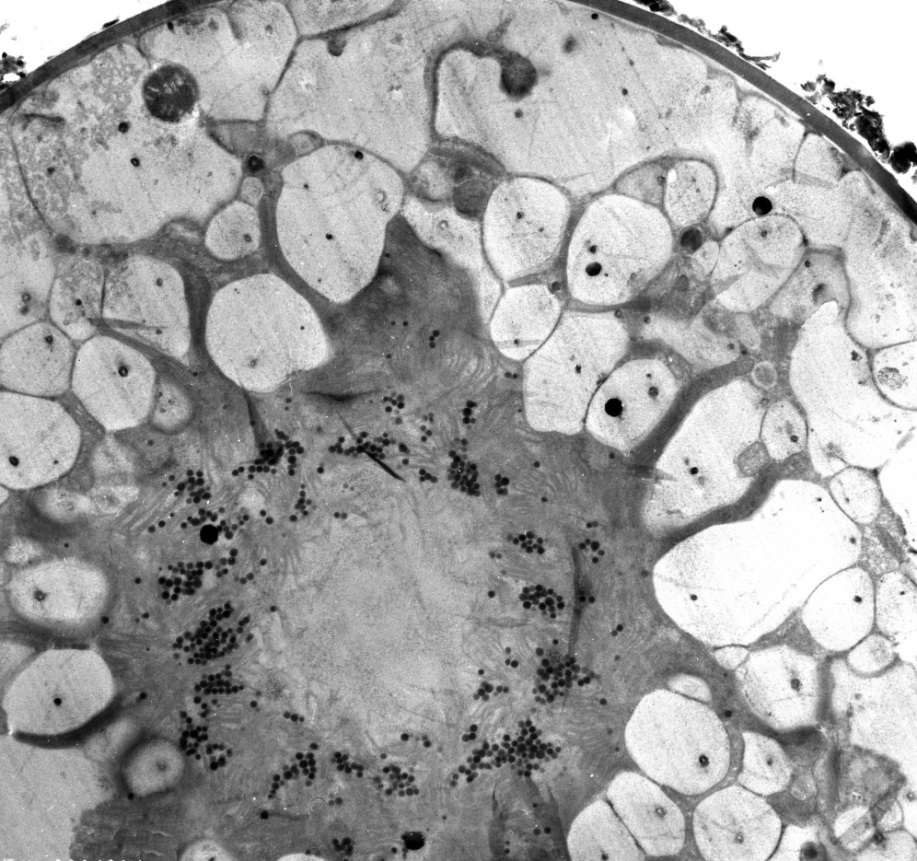


**L**

**C**

**P**

**Supplementary Figure 4.** TEM (left) and LM (right) micrograph of *Cd.* cf. *nivalis* collected from red snow in Tyrol, Austrian Alps. Note the typical cryoconite particles frequently attached to the cell walls. Abbreviations: L, lipid body; C, chloroplast (with many electron-dense plastoglubules); P, ‘naked’ pyrenoid. Bar: 1 *µ*m (TEM), 10 *µ*m (LM).

0,0

5,0

10,0

15,0

20,0

25,0

30,0

35,0

40,0

45,0

min

0

25

50

75

100

125

150

175

200

225

250

275

mAU

DR53.lcd 480 nm

**Supplementary Figure 5.** Typical HPL-chromatogram of *Chlainomonas* sp. (sample DR53) at 480 nm. Two groups of peaks are indicated: Astaxanthin-monoesters (red dashed), astaxanthin-diesters (green dashed). The amount of diesters (total peak area) in this species is always larger than that of monoesters.

300

400

500

600

nm

0

50

100

150

200

250

300

mAU

2,163

475

300

400

500

600

nm

0

5

10

15

20

25

30

35

mAU

2,570

466

370

**Supplementary Figure 6.** HPLC online spectral absorptions of all *trans*-astaxanthin standard (left) and of *13Z cis-*astaxanthin standard (right). Peaks with these spectra were found in both *Chloromonas* sp. and *Cd.* cf. *nivalis*.


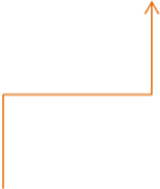

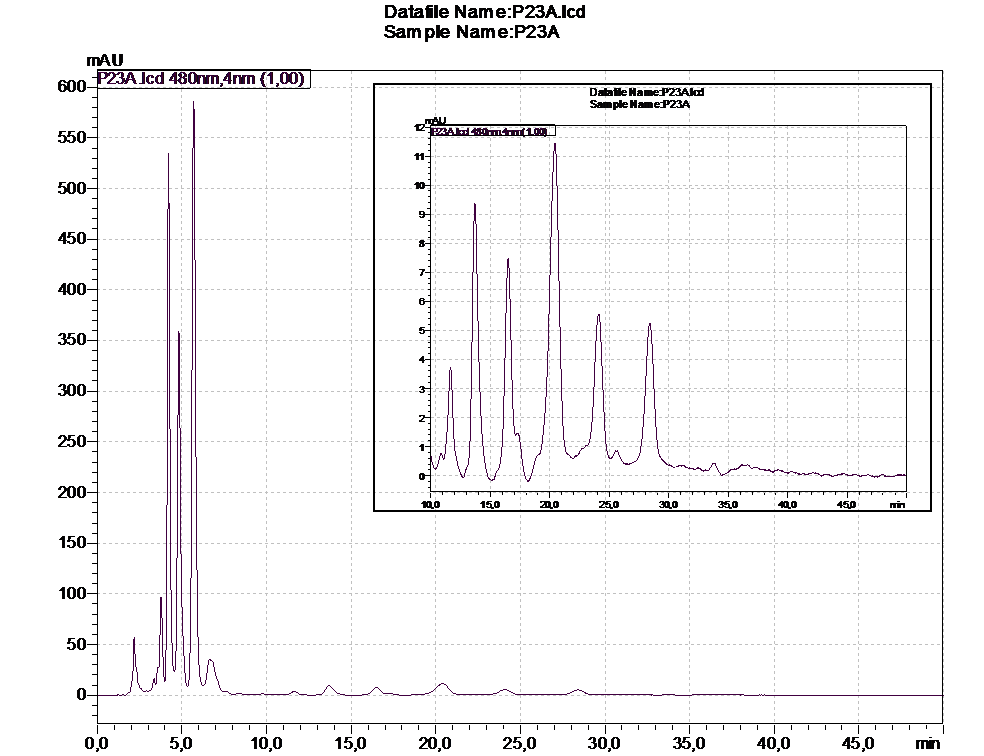


**Supplementary Figure 7.** Representative HPL-chromatogram of *Chlamydomonas* cf. *nivalis* (sample DR23) at 480 nm, showing the distribution of astaxanthin-derivative peaks*.* Red frame: monoester peaks, green frame: diester peaks; inset (marked with orange arrow): enlargement of diester peak region. In this species, the total amount of diesters (summed peak area) is always lower than monoesters.


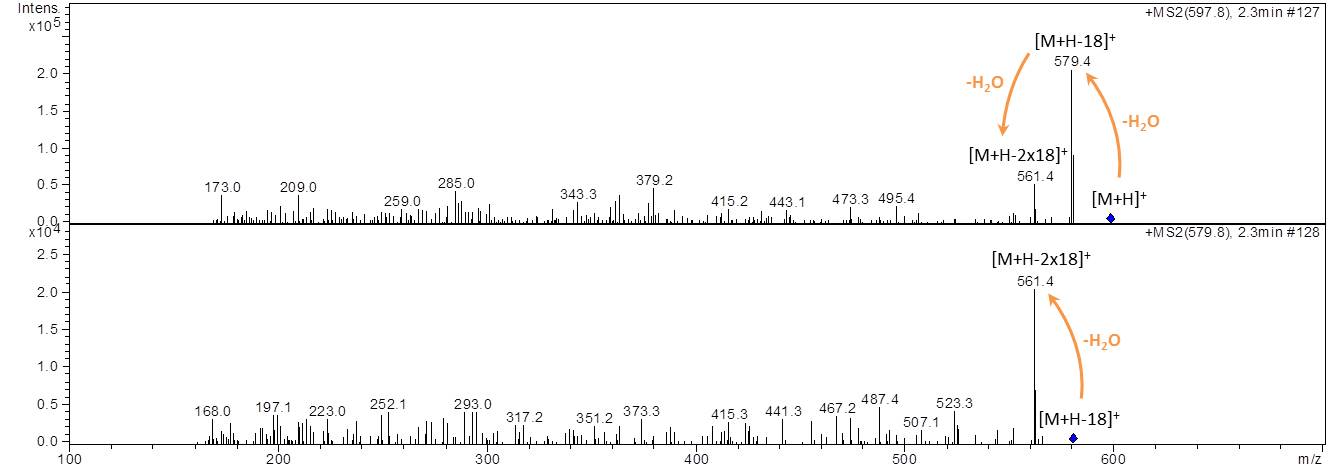


**Supplementary Figure 8.** Typical pattern of LC-MS compound fragmentation (MSMS^+^ mode) of an astaxanthin standard (retention time: 2.3 min, *m/z* = 597.8) with reduction of one, respectively two water molecules [M+H-(2x)18]. The second spectrogram shows the active fragmentation of the first fragment, again water is cleaved off from astaxanthin.


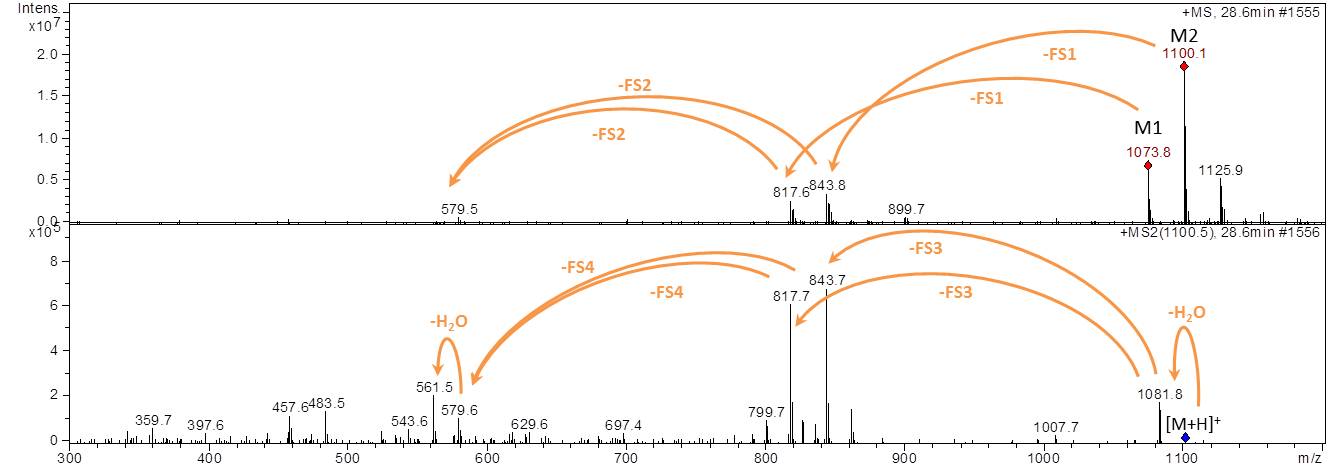


**Supplementary Figure 9.** Spectrograms of typical spontaneous (above) and active (below) MS fragmentation of two different astaxanthin-diesters (M1 and M2) of *Chlainomonas* sp. occurring at retention time 28.6 min. The putative way of fragmentation and fragments are indicated with arrows. (FS, fatty acid).

**Supplementary Video 1.** Presentation of the slow but active movement of a quadriflagellate swarmer of *Chlainomonas* sp. (sample HW02), observed in a microscope specimen chamber thermostated to 1°C. [Suppl_Video_Chlainomonas_HW02]
